# Evolution of bacteria in the human gut in response to changing environments: An invisible player in the game of health

**DOI:** 10.1016/j.csbj.2021.01.007

**Published:** 2021-01-11

**Authors:** Aarushi Venkatakrishnan, Zoie E. Holzknecht, Rob Holzknecht, Dawn E. Bowles, Sanet H. Kotzé, Jennifer L. Modliszewski, William Parker

**Affiliations:** a Department of Surgery, Duke University School of Medicine, Durham, NC, USA; b Department of Biomedical Sciences, Faculty of Medicine and Health Sciences, University of Stellenbosch, Cape Town 8000, South Africa; c Genomic Analysis and Bioinformatics Shared Resource, Duke Center for Genomic and Computational Biology, Duke University School of Medicine, Durham, NC, USA

## Abstract

Several factors in Western society, including widespread use of antibiotics, chronic inflammation, and loss of complex eukaryotic symbionts such as helminths, have a dramatic impact on the ecosystem of the gut, affecting the microbiota hosted there. In addition, reductions in dietary fiber are profoundly impactful on the microbiota, causing extensive destruction of the niche space that supports the normally diverse microbial community in the gut. Abundant evidence now supports the view that, following dramatic alterations in the gut ecosystem, microorganisms undergo rapid change via Darwinian evolution. Such evolutionary change creates functionally distinct bacteria that may potentially have properties of pathogens but yet are difficult to distinguish from their benign predecessors.

## 1 Introduction: Evolution of the gut microbiota and health

The view that a healthy gut microbiota is necessary for the optimal functioning of the human intestinal tract, thereby influencing overall health and wellbeing, is now widely accepted. Perhaps the most striking and obvious illustration of this view is a condition known as recurrent *Clostridium difficile* colitis. This condition occurs when the gut microbiota is destabilized by medical use of antibiotics, after which the organism *Clostridium difficile*, often present in very low levels, becomes a prominent microorganism in the gut. The condition is potentially lethal, and often difficult to manage without a fecal transplant from a donor with a healthy gut microbiota.

Fecal therapy, in which feces from a healthy donor is transferred to patients with gastrointestinal problems, is often successful for treating conditions other than *C difficile* colitis [1], [2], [3]. However, in many of these cases, it is not possible to determine what exactly was wrong with the patient’s gut microbiota in the first place. Some organisms can be found that are associated with disease, but it remains unknown if this is cause or effect [3], [4], and associations are not consistent [4]. Thus, an unhealthy gut microbiota is often difficult to identify [4], and mystery surrounds exactly where such a microbiota may have originated.

In this paper, we will consider biological evolution, Darwinian selection, as a driving force for changes to the microbiota in the gut. We will argue that these evolutionary changes inevitably follow any significant change in the gut ecosystem, and that numerous changes in the gut caused by Western lifestyles will dramatically accelerate the evolutionary processes naturally occurring in the microbiota. Finally, we demonstrate that the “evolutionary time” present in the gut based on the number of organisms and their reproduction rate is almost incomprehensibly vast when compared side-by-side with the evolutionary potential for vertebrate species such as humans. Indeed, experimental evidence, summarized in this review, points to the idea that significant evolutionary change in the gut microbiota can occur in a matter of a few weeks.

The view that evolutionary change can reshape the gut microbiota has profound implications for digestive disorders such as inflammatory bowel disease infla and celiac disease, and for our efforts to track disease associated organisms. This view in fact suggests that the human gut can be the source of its own pathogenic bacteria. Under such circumstances, controlling factors that affect the niche space of the gut may be the only effective means of preventing the emergence of potentially harmful bacteria via evolutionary processes. Further, subtle evolutionary changes in the microbiota help provide an explanation for the observation that transplantation of feces from healthy donors is often a viable method of treating sick patients, despite the fact that the underlying problems with the microbiota in sick patients might not be evident based on species composition.

## 2 Fiber and niche space in the gut

Organisms evolve in a manner that allows them to successfully occupy niche space in their environment. In the gut of omnivores including humans and laboratory rodents, numerous lines of evidence, reviewed by Pereira and Berry [5], strongly point toward dietary fiber as being the primary factor that creates and defines niche space in the gut. Dietary fibers vary widely in structure and provide a rich source of energy for numerous groups of bacteria. But with a Western diet, fiber intake is much lower and microbes in the gut are forced to compete for limited resources [6]. An elegant illustration of this competition for limited resources in the absence of fiber can be observed in mice containing only one bacterial species. Wu and colleagues found that survival of *Bacteroidetes cellulosilyticus* in a gnotobiotic mouse model depended on 16 times more loci (550 versus 34 total loci) when their hosts were fed a high-fat, high-sugar diet than when their hosts were fed a low-fat, high-plant polysaccharide diet [7]. In this experiment, without fiber, microbes were genetically constrained, with mutants unable to find space in the environment for survival. In contrast, in the presence of fiber, mutants specialized in the digestion of particular fiber structures, allowing for microbial diversity to emerge.

## 3 Modern society changes the niche space in our gut

The microbiome has experienced numerous changes due to Westernization. For example, an average of approximately 2.5 doses of antibiotics are consumed per every 100 people *on any given day* in Western countries [8]. Dramatic changes in the microbiota community composition as a result of antibiotics have been studied extensively [9], [10]. In addition, essentially all complex eukaryotic symbionts, including helminths and protists, have been eliminated by sanitation technology from the human gut ecosystem in Western countries [11], and this loss of species diversity may also have a significant impact on the microbiota community composition [12], [13]. Whether by sanitation or by antibiotics, elimination of species from the gut ecosystem induces a change in niche space utilization by remaining species. Not only are niche spaces left vacant following loss of biodiversity, but niche spaces that are dependent on the presence of particular species can disappear.

Further, chronic inflammation of the gut associated with inflammatory bowel diseases and other digestive disorders such as celiac disease have become increasingly common in Western society [14], [15], [16], potentially altering the interaction between the host immune system and the microbiota [17]. The presence of chronic inflammation presents the disturbing specter of widespread, across-the-ecosystem alteration of niche space in the gut that is centered around the immune-mediated communication between host and symbionts.

**Fig. 1:**
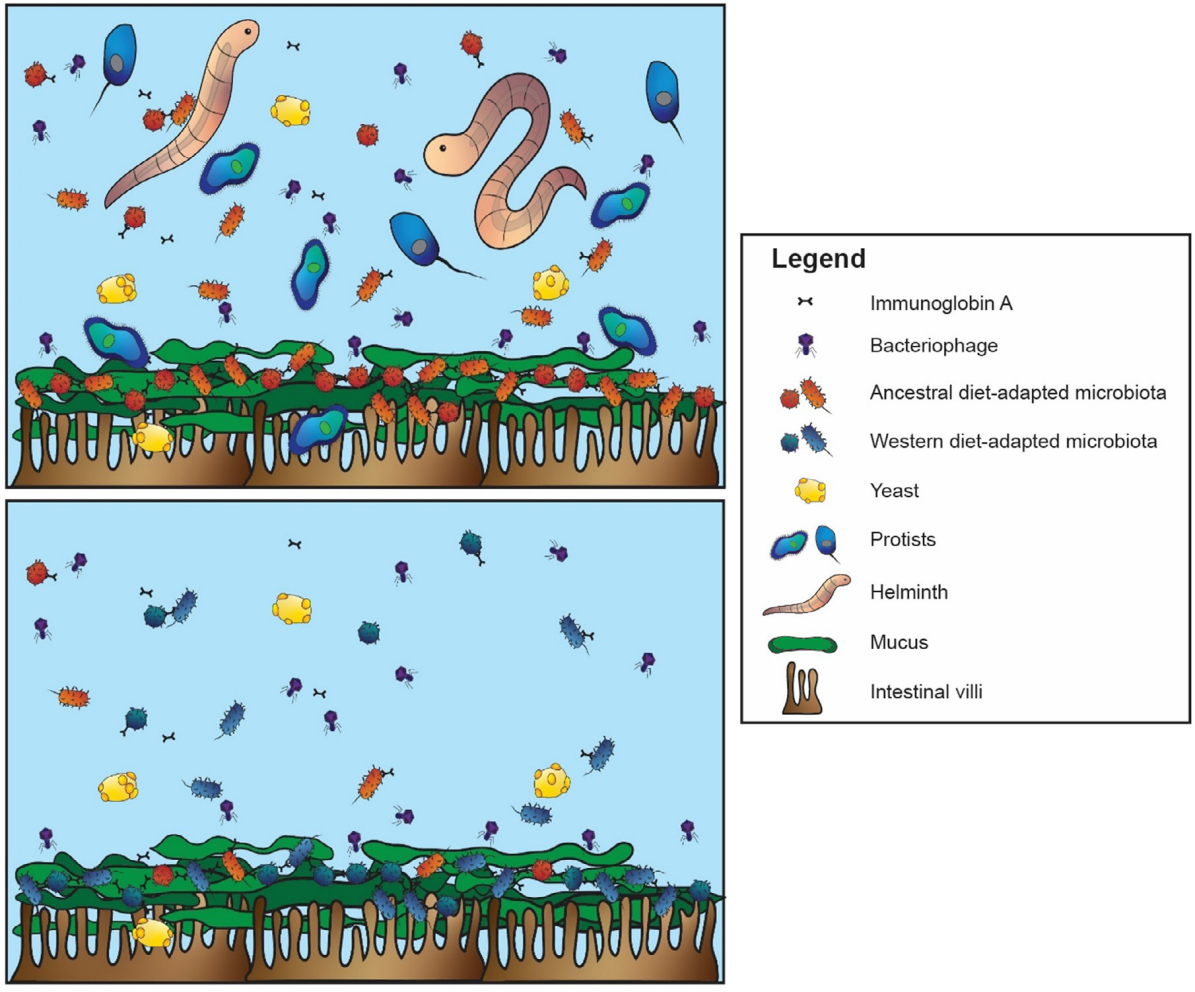
An illustration of the hunter-gatherer gut biome (A) compared with the gut biome of a typical Westerner (B). Both ecosystems contain a wide range of microorganisms, including phages, bacteria, and yeast. However, the Western biome lacks helminths and protists, and has a microbiota altered by a dramatic decrease in dietary fiber and increased chronic inflammation, among other issues.

An illustrative diagram of the changing gut is shown in Fig. 1. Not only are complex eukaryotic symbionts absent, but the microbial communities are substantially altered due to changes in fiber consumption [18], [19] and perhaps in more subtle ways by chronic inflammation [20], [21]. Given that, as described above, fiber is a major factor that defines niche space in the gut, it is unsettling that fiber consumption has changed so much in the Western diet. Many of the common foods in the Western diet are very low in fiber or even fiber free. For example, all meat-based foods, such as beef, poultry, and fish have no fiber. Further, unlike human breast milk, dairy-based products are almost completely lacking in carbohydrates that nourish the gut flora [22], [23]. Further, a variety of other foods such as rice-based cereals, cakes, and white breads have little to no fiber. In general, the common use of food processing in Western culture creates a more desirable food to the uneducated human palate, but removes much of the fiber.

When observing the fiber intake of Western people and non-Western groups in rural Africa during the late 1960s/early 1970s, Denis Burkitt found that Westerners had modified their diet in such a way as to dramatically reduce fiber consumption [24], [25]. In 2019, it was observed that people in Western societies consume about 66% less fiber compared to those in rural South Africa and Uganda [26]. Pontzer and colleagues estimate that the Hadza population, a hunter-gatherer society in northern Tanzania, have a daily fiber intake of 80–150 g, which is 4 to 7.5 times greater than the 20 g of daily fiber intake found for the average person in the US [27]. Eaton and colleagues came to similar conclusions, finding that the late paleolithic diet contains about 100–150 g of fiber/day, whereas the contemporary American diet contains only 19.7 g of fiber/day [28]. Combined, it can be estimated that the US diet experiences a 75–92.5% fiber loss compared to that of hunter-gatherers. Especially concerning is the extremely low fiber intake by a fraction of American individuals [29].

## 4 The effects of changing fiber consumption on the gut microbial community composition

**Fig. 2:**
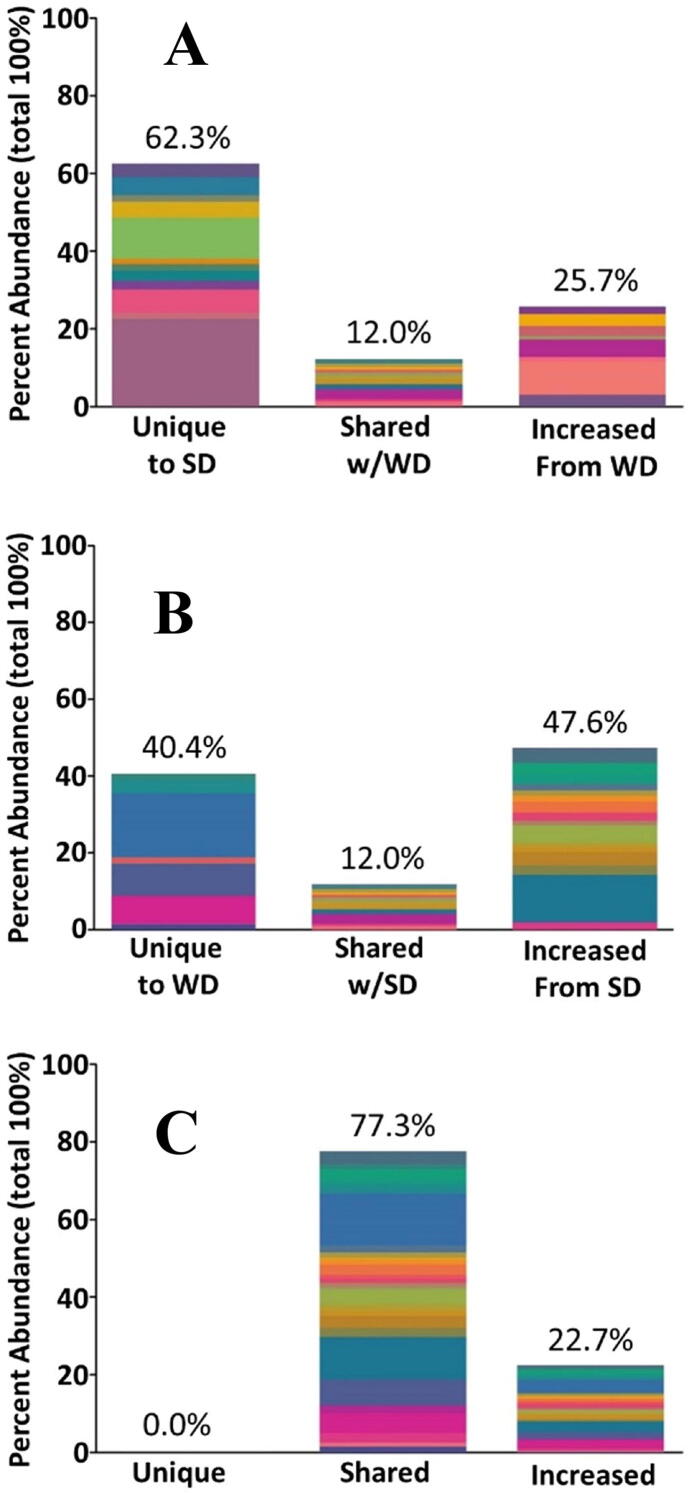
Comparison of fecal microbiota community comparison between (A) mice fed a Standard Diet with fiber (SD, n = 4) and (B) mice fed a Western diet without fiber (WD, n = 10). Data were derived from the study reported by Hyoju et al (30), and each color represents a specific amplicon sequence variant (ASV). ASVs were filtered to include only the most prevalent ASVs. ASVs in a given microbial community were placed in two categories which add up to a total of 100%: ASVs that are unique to a given group and ASVs that co-occur in the two diet groups. The relative abundance of ASVs that co-occur was further divided into the proportion of variation that is shared between the two groups (“Shared”) and the remainder (“Increased”), which is found in one of the two groups. For comparison purposes, 100 random sets of two groups of five mice were created within the Western Group and the relative proportions of ASVs within the two groups were calculated (C). Less than 15% of the microbiota remains the same (shared) when comparing a Standard diet to a Western diet. In contrast, in the 100 random samplings of Western diet into two groups, on average 75% of their microbiota is shared. Methods used in the analysis were as follows: Fastp (v0.20.1) was used to verify that reads were adaptor-free. The fastx_quality_stats tool from Fastx-toolkit (v0.0.14) was used to determine median base quality for each position of the reads for each region. Reads were imported into qiime2^3^ (v2020.2), and denoised and dereplicated with dada2 (via q2-dada2). In dada2, reads were trimmed at the beginning or truncated at the end if the median base quality fell below a score of 30 as determined by Fastx-toolkit. Taxonomy was assigned to ASVs using the q2‐feature‐classifier classify‐sklearn naïve Bayes taxonomy classifier against the SILVA 132 database. ASVs identified as uncharacterized, mitochondrial, chloroplast, or Eukaryota were filtered from the dataset, and samples with fewer than 1000 reads were also excluded. All remaining ASVs were aligned with Mafft (via q2‐alignment, v.7.310) and used to construct a phylogeny with Raxml version 8(via q2‐phylogeny). ASVs were filtered to exclude any ASVs that were not observed in at least two samples overall and did not have a relative abundance of at least 1% across either all Western diet or all standard diet samples.

Perhaps surprisingly, few studies in laboratory animals have quantitatively probed the effects of loss of fiber on the microbiota. One notable exception to this is a study by John Alverdy’s group in Chicago, who evaluated the effects of removing fiber from the diets of laboratory mice [30]. Their results demonstrated clearly that antibiotic treatment of the microbiota, as expected, changed the community composition of the microbial community in the gut. However, the microbial community in mice fed a normal rodent diet that includes fiber recovered rapidly from antibiotic treatment, rebounding quickly back to the community composition that existed prior to treatment. The perhaps unexpected finding was that the microbial community in mice fed a diet without fiber failed to recover from antibiotics. This result provides an excellent illustration of how rapid alteration of niche space in an ecosystem can destabilize the life inhabiting that ecosystem. A quantitative illustration of the effects of fiber loss in Alverdy’s mice is shown in Fig. 2. Following loss of dietary fiber, approximately 85% of the community structure is changed due to the presence of new species of bacteria or due to changes in the abundance of previously existing species. This observation demonstrates that loss of dietary fiber causes very profound and potentially catastrophic upheavals in the microbial community, which in turn are indicative of substantial alterations in the niche space of the gut.

## 5 Evolution of the gut microbiota following environmental changes

**Fig. 3:**
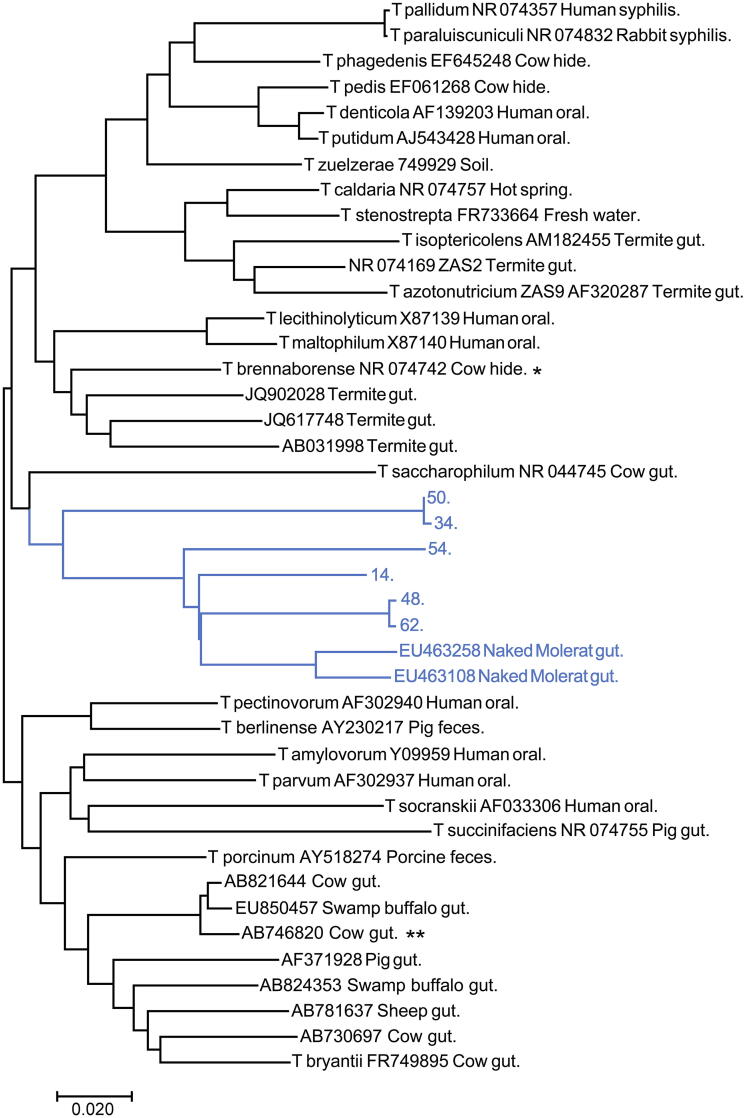
*Treponema* phylogenetic tree constructed with full length 16S rRNA sequences. Sequences from the mole-rat are shown in light color, with nearest neighbors in black. The evolutionary history was inferred using the Neighbor-Joining method [1]. Sequences were aligned with CLC Sequences Viewer or with MUSCLE. (Previously published trees (35) using sequences aligned with SSU-ALIGN are less parsimonious.) The optimal tree with the sum of branch length = 1.87461316 is shown. The tree is drawn to scale, with branch lengths in the same units as those of the evolutionary distances used to infer the phylogenetic tree. The evolutionary distances were computed using the Maximum Composite Likelihood method [2] and are in the units of the number of base substitutions per site. The analysis involved 42 nucleotide sequences. All positions containing gaps and missing data were eliminated. There were a total of 1248 positions in the final dataset. Evolutionary analyses were conducted in MEGA7 [3]. *Sequence with nearest identity to a sequence from mole-rat gut, used for presumptive identification of clade. **Sequence with nearest identity to sequences from mole-rat gut (from an unknown bacterial species and therefore not used for identification of clade).

Rapid shifts in niche space generally induce rapid changes in microbial community composition. In pre-agrarian human cultures, for example, seasonal changes in diet correspond to periodic changes in microbial community composition [31]. Such changes apparently involve transitions between well-established and stable microbial community compositions, and might be considered as akin to reproducible transitions that occur during the weaning of mammals [32]. In contrast, novel, dramatic changes in niche space are often followed by extinction of existing species and subsequent rapid evolutionary changes of remaining species [33], [34]. Because, as described above, modern society affects key factors such as diet and chronic, pathologic inflammation that define niche space in the gut, dramatic evolutionary changes may be expected in the microbial communities of humans living in modern society. These evolutionary changes require random mutation and the emergence of new phenotypes, a process very distinct from shifts in microbial community composition that are rapidly observed. An illustration of Darwinian evolution in the gut microbiota following a change in diet has been observed in the gut of the African mole-rat [35]. Mole-rats have an extremely cellulose-rich diet compared to other rodents, providing an opportunity to evaluate the effects of an increase in a particular dietary fiber on the microbiota. For the evaluation, genetic sequences obtained from the bacteria of mole-rats have been compared with sequences of related bacteria from a wide range of species [35]. The results showed that microbial species associated with cellulose digestion had evolved extensively in the mole-rat gut. For example, as shown in Fig. 3, substantial genetic differences between spirochetes from mole-rats and spirochetes from other species were observed, suggesting that cellulose digesting bacteria in the mole-rat gut had undergone extensive evolution-driven changes. In contrast, bacteria in the mole-rat gut not known to be associated with the digestion of cellulose showed little change compared to bacteria found in other species [35]. The data suggest that some of the cellulose-digesting bacteria in the mole-rat are most closely related to bacteria found in modern cattle, although they had diverged substantially from those bacteria.

The microbiota of the African mole-rat have evolved for tens of millions of years following the divergence of their hosts from other rodents. Such long-term evolutionary processes may be of little concern during a single human lifetime, but much more rapid examples of evolution of the gut microbiota can be seen. For example, when a single isolate of *Escherichia coli* was introduced into a previously bacteria-free mouse colony, substantial changes were observed within a few weeks [36]. Continued monitoring revealed ongoing changes over the subsequent two to three years until the experiment was terminated [36]. Evolution of the laboratory bacteria was profound, as the single isolate diverged into a community of organisms with variable metabolic profiles, variable sizes, enhanced ability to survive in the gut environment, and a diminished ability to survive in a laboratory environment [36].

## 6 Opportunities for evolution due to an abundance of microbial reproduction in the gut

The speed at which a given species has the capacity to evolve is a complex function of the number of offspring produced per unit time, the total number of species present at any time, and numerous complexities associated with their genetics and reproduction [33]. Thus, it is exceedingly difficult to accurately estimate capacity for evolutionary change for a species. Furthermore, objective measures of evolutionary change are probably not applicable across a wide range of species. Nevertheless, the rate of reproduction can be taken as one very simple indicator of the capacity of a species to evolve, with more reproductive events equating to more capacity to evolve. With this in mind, for the sake of creating an intuitive side-by-side comparison, it is possible to estimate the reproductive rate of a species familiar to us (ourselves; *Homo sapiens*) alongside the reproductive rate of the bacteria in our own gut. For the sake of this comparison, the entire human population prior to the advent of agriculture can be considered alongside the gut microbiota of a single individual. Based on values provided in the literature regarding the amount of feces produced by a human and the amount of bacteria in a given amount of feces [37], [38], it can be estimated that, in a given day, approximately 1.1 × 10^13^ bacterial cells are produced in an average human gut. In contrast, given estimates regarding the total number and reproduction of hunter gatherers prior to the agricultural revolution, with approximately 5 million females, and approximately 4.4 offspring in a generation time of 27 years [39], [40], it seems likely that less than 2,250 humans were born each day prior to the agricultural revolution.

**Fig. 4:**
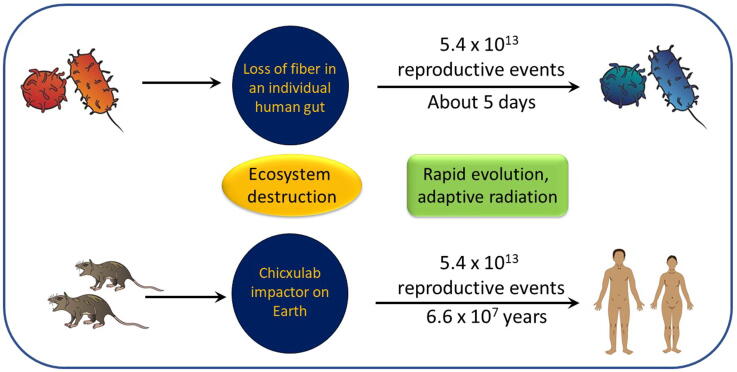
A relative comparison of reproductive events per unit time between the microbiota in one human gut (top) versus the entire human population prior to the development of agriculture (bottom). For illustrative purposes, the loss of dietary fiber is compared with the Chicxulub impactor that occurred an estimated 66 million years ago. Importantly, no attempt was made to estimate the number of reproductive events spanning the evolution of modern humans from early mammals during the 66-million-year time span. Rather, the number of reproductive events was calculated based on the estimated reproductive rate of hunter-gatherers prior to the agricultural revolution as described in the text. Further, no attempt was made to calculate reproductive events for individual species of bacteria in the gut. Rather, the microbiota as a whole was considered in the calculations.

Given that the average human lifespan in the US in 2015 was about 79 years [41], approximately 3.16 × 10^17^ bacteria are produced in the average human lifetime of a single human (ignoring age-dependent factors, among other issues). For hunter-gathers prior to the agricultural revolution to have that many reproductive events would take approximately 3.88 × 10^11^ years, or approximately 28 times the age of our universe. Although this interesting comparison is not meant to be precise, it does illustrate the point shown in Fig. 4: the number of reproductive events that happen in our gut in less than a week exceeds the number of reproductive events that could occur in tens of millions of years of human evolution in hunter-gatherers. The bottom line is that the human gut harbors a tremendous number of reproductive events, which equates to greater potential to adapt to a changing environment.

## 7 Conclusions

Changes in the ecosystem of our gut as a result of Western society have destabilized microbial communities in the gut. Foremost among these changes are a loss of fiber from the Western diet and the emergence of widespread, chronic inflammation of the gut associated with disease. In addition, the frequent use of antibiotics and the loss of complex eukaryotic symbionts contribute to changes in the gut ecosystem. As the environment is disturbed, the vast capacity of the gut microbiota for Darwinian evolution will inevitably lead to altered organisms in the gut. In this manner, it is possible that we can create or own disease-adapted, potentially pathogenic microbes. Given that the microbiota is the only part of our body that is passed from generation to generation directly through transmission and not through reproduction, this insight is particularly concerning.

Important factors emerge from this discussion. First, evolution of the microbiota following a change in the gut environment is probably unavoidable. Second, this evolution can happen rapidly, resulting in significant changes within a matter of weeks. It has been proposed that inflammation in the gut as a consequence of disease may lead to the evolution of bacteria adapted to inflammation and the disease state [42]. Unfortunately, these bacteria may in turn help stabilize the disease state, leading to persistence of that state and resistance to medical treatment [42].

Microbiologists have long seen the gut microbiota as a complex community of organisms that can be characterized and defined. Although host species do have propensities for particular microbial community compositions [43], the definition of a healthy core microbiota in humans is challenging to define [44]. Adding the dimension of time and evolution to the system adds a vast complexity on top of an already extremely complex system. Nevertheless, Darwinian evolution of the microbiota in the gut occurs rapidly and inevitably following novel changes to the gut environment. Although it remains unknown at the present time to what extent evolution of the microbiota affects the disease state, such evolution may account for difficulty in identifying associations between specific disease states and bacterial species. Further, evolution of the microbiota in a manner adapted to an inflammatory state could account for the fact that fecal transplants from healthy donors often work in patients with inflammatory disorders, despite the fact that no microbial species can be identified as responsible for those disorders. Finally, with these considerations in mind, Darwinian evolution of the microbiota within their hosts remains a largely unexplored field of research that is potentially critical for understanding the disease state of the intestine.

## CRediT authorship contribution statement

**Aarushi Venkatakrishnan:** Data curation, Formal analysis, Investigation, Methodology, Project administration, Writing - original draft. **Zoie E. Holzknecht:** Data curation, Formal analysis, Investigation, Methodology, Project administration. **Rob Holzknecht:** Data curation, Formal analysis, Investigation, Methodology, Project administration, Resources, Software, Supervision, Validation, Visualization. **Dawn E. Bowles:** Data curation, Formal analysis, Funding acquisition, Investigation, Methodology, Project administration, Resources. **Sanet H. Kotzé:** Funding acquisition, Investigation, Methodology, Project administration, Resources. **Jennifer L. Modliszewski:** Data curation, Formal analysis, Writing - original draft. **William Parker:** Data curation, Formal analysis, Funding acquisition, Investigation, Methodology, Project administration, Resources, Software, Supervision, Validation, Visualization, Writing - original draft.

## Declaration of Competing Interest

The authors declare that they have no known competing financial interests or personal relationships that could have appeared to influence the work reported in this paper.
